# Occupational Therapy Students’ Evidence-Based Practice Skills as Reported in a Mobile App: Cross-Sectional Study

**DOI:** 10.2196/48507

**Published:** 2024-02-21

**Authors:** Susanne G Johnson, Birgitte Espehaug, Lillebeth Larun, Donna Ciliska, Nina Rydland Olsen

**Affiliations:** 1 Department of Health and Functioning Western Norway University of Applied Sciences Bergen Norway; 2 Division of Health Services Norwegian Institute of Public Health Oslo Norway; 3 Faculty of Health Sciences McMaster University Hamilton, ON Canada

**Keywords:** active learning strategies, application, cross-sectional study, development, education, higher education, interactive, mobile application, mobile app, occupational therapy students, occupational therapy, students, usability, use

## Abstract

**Background:**

Evidence-based practice (EBP) is an important aspect of the health care education curriculum. EBP involves following the 5 EBP steps: ask, assess, appraise, apply, and audit. These 5 steps reflect the suggested core competencies covered in teaching and learning programs to support future health care professionals applying EBP. When implementing EBP teaching, assessing outcomes by documenting the student’s performance and skills is relevant. This can be done using mobile devices.

**Objective:**

The aim of this study was to assess occupational therapy students’ EBP skills as reported in a mobile app.

**Methods:**

We applied a cross-sectional design. Descriptive statistics were used to present frequencies, percentages, means, and ranges of data regarding EBP skills found in the EBPsteps app. Associations between students’ ability to formulate the Population, Intervention, Comparison, and Outcome/Population, Interest, and Context (PICO/PICo) elements and identifying relevant research evidence were analyzed with the chi-square test.

**Results:**

Of 4 cohorts with 150 students, 119 (79.3%) students used the app and produced 240 critically appraised topics (CATs) in the app. The EBP steps “ask,” “assess,” and “appraise” were often correctly performed. The clinical question was formulated correctly in 53.3% (128/240) of the CATs, and students identified research evidence in 81.2% (195/240) of the CATs. Critical appraisal checklists were used in 81.2% (195/240) of the CATs, and most of these checklists were assessed as relevant for the type of research evidence identified (165/195, 84.6%). The least frequently correctly reported steps were “apply” and “audit.” In 39.6% (95/240) of the CATs, it was reported that research evidence was applied. Only 61% (58/95) of these CATs described how the research was applied to clinical practice. Evaluation of practice changes was reported in 38.8% (93/240) of the CATs. However, details about practice changes were lacking in all these CATs. A positive association was found between correctly reporting the "population" and "interventions/interest" elements of the PICO/PICo and identifying research evidence (*P*<.001).

**Conclusions:**

We assessed the students’ EBP skills based on how they documented following the EBP steps in the EBPsteps app, and our results showed variations in how well the students mastered the steps. “Apply” and “audit” were the most difficult EBP steps for the students to perform, and this finding has implications and gives directions for further development of the app and educational instruction in EBP. The EBPsteps app is a new and relevant app for students to learn and practice EBP, and it can be used to assess students’ EBP skills objectively.

## Introduction

Evidence-based practice (EBP) involves using the best available evidence from relevant research and integrating it with clinical expertise, patient values, and circumstances to make clinical decisions for individual patients [1]. When applying EBP, it is recommended to follow the five EBP steps: (1) identifying information needs and formulating answerable questions (ask), (2) finding the best available evidence to answer clinical questions (assess), (3) critically appraising the evidence (appraise), (4) applying the results in clinical practice (apply), and (5) evaluating performance (audit) [1,2]. These 5 steps reflect the suggested core competencies covered in teaching and learning programs to support future health care professionals applying EBP, including developing EBP knowledge and skills [3].

EBP skills can be understood as applying EBP knowledge by performing EBP steps, ideally in a clinical setting [4]. The literature indicates that EBP knowledge and skills improve when EBP teaching and learning are multifaceted, interactive, clinically integrated, and incorporate assessment [5]. When implementing EBP teaching, it is relevant to document and assess the individual student’s performance [3,5,6]. As it is recommended to follow all 5 EBP steps when teaching and learning EBP [1,2], measuring the performance of all 5 steps is relevant when evaluating EBP learning. However, few evaluation instruments measure all 5 EBP steps [5-9], and most instruments are self-reported questionnaires [6,7]. The use of self-reported questionnaires may contribute to biased results due to recall bias or social desirability responses [9,10]. Objectively measuring EBP learning could result in a true reflection of the situation, and thus, it is recommended to develop objective tools for EBP learning assessment [6,7,11]. To objectively document the performance of the EBP steps, Shaneyfelt et al [6] emphasized using online documentation. Online documentation is feasible through mobile apps, and innovative new methods to evaluate EBP teaching can now be explored [12]. Most students own a smartphone, which makes mobile learning and information sharing possible [13,14]. Thus, mobile apps can potentially be used for documenting and assessing students’ EBP performance. The aim of this study was to assess occupational therapy (OT) students’ EBP skills as reported in a mobile app.

## Methods

### Design

This study used a cross-sectional design. The reporting of this study followed the STROBE (Strengthening the Reporting of Observational Studies in Epidemiology) checklist (Multimedia Appendix 1) [15].

### Mobile App

A mobile web app called the EBPsteps app was developed at the Western Norway University of Applied Sciences (HVL) to support health and social care students’ EBP learning [16]. An updated version of this web app is now freely available as a native app [17]. Experiences with using the EBPsteps app for learning EBP have previously been explored [16]. The app provides an opportunity for students to document the 5 EBP steps. A description of the content of the EBPsteps app is presented in Textbox 1.

**The EBPsteps app content.**

**Ask**

- Reflect on information needs
- Formulate the clinical question
- Identify the type of clinical question (drop-down menu)
- Identify the Population, Intervention, Comparison, and Outcome/Population, Interest, and Context (PICO/PICo) elements

**Assess**

- Report information source used to identify research evidence
- Report links to research evidence identified

**Appraise**

- Choose a relevant critical appraisal checklist
- Complete the critical appraisal using the integrated checklist

**Apply**

- Report how research evidence was applied in practice (drop-down menu)

**Audit**

- Report if changes in practice were completed and evaluated
- Describe changes if changes were implemented
- Evaluate the EBP process (ask, assess, appraise, apply, and audit)

By documenting the EBP process in the app, students produced critically appraised topics (CATs). A CAT can be explained as a summary of research evidence on a clinical question [18]. The CATs completed in the EBPsteps app included information on all EBP steps, and the CATs could be sent through email and shared as a PDF document. The CATs produced in the app were stored on the HVL research server and were accessible to the researchers in this project.

### Participants

A total of 4 cohorts of fifth-semester OT students from different academic years (from 2018 to 2021) at HVL were eligible for inclusion if they used the EBPsteps app.

### Setting

In Norway, OT education is a 3-year bachelor’s degree of 6 semesters (180 European Credit Transfer System [ECTS]). According to the Norwegian national curriculum, all health and social care students must be able to acquire new knowledge and make professional assessments, decisions, and actions in line with EBP [19]. At the time of this study, EBP was well integrated into the OT bachelor’s degree program at HVL [20].

Textbox 2 provides an overview of the total number of standalone EBP sessions (n=27) that OT students in this study received by their fifth semester (year 3). This amount of EBP teaching hours is a high number [21]. In addition, EBP was integrated into other learning activities, such as problem-based learning (PBL) group activities, written assignments, and exams.

Using the EBPsteps app was part of the EBP teaching. Students were introduced to the app at the start of the fifth semester. The students watched a video presentation of how to use the app and explored using the app while being supervised by a teacher. During the fifth semester, the students were encouraged to use the EBPsteps app on campus (4 weeks) and during clinical placements (11 weeks). While on campus, students had to use either the EBPsteps app or a Microsoft Word document to complete a mandatory EBP assignment that involved producing a CAT on a clinical topic. Similarly, at the end of the semester, an appendix to the home exam was to use either the EBPsteps app or a Word document to produce a CAT.

**Overview of standalone EBP sessions. Year 3 includes sessions given through the fifth semester only. EBP: evidence-based practice.**

**Year 1**

- Standalone sessions about “ask” (2 hours) and “assess” (2 hours). Total duration is 4 hours.

**Year 2**

- Standalone sessions about “ask” (1 hour), “assess” (1 hour), “appraise” (3 hours), and “apply” (2 hours). Total duration is 7 hours.

**Year 3**

- Standalone sessions about “ask” (2 hours), “assess” (2 hours), “appraise” (8 hours), “apply” (3 hours), and “audit” (1 hour). Total duration is 16 hours.

### Data Collection

CATs produced by students during the fifth semester were exported from students’ user accounts in the EBPsteps app to Microsoft Excel [22] at the end of the semester. The Norwegian data, anonymized by authors, are freely available through HVL Open [23] and include our assessment. To objectively assess students’ EBP skills based on how they documented the EBP process in the app, we developed a scoring plan for each EBP step in the CATs (Multimedia Appendix 2). The different steps of the CATs were assessed as correct or incorrect, which were the outcomes investigated in this study. Two researchers independently scored each CAT, and disagreements were resolved through discussion. An overview of the scoring plan is presented in Textbox 3.

**Overview of the scoring plan. Includes the EBP steps and what was assessed. EBP: evidence-based practice.**

**Ask**

- Was it reflected on the information needs?
- Which clinical question was formulated (eg, prevalence, cause, diagnostics, effect of measures, prognosis, or experiences and attitudes)?
- Which clinical question was identified (drop-down menu)?
- Was there an agreement between the formulated clinical question and the type of question identified from the drop-down menu?
- Was the “population” of the Population, Intervention, Comparison, and Outcome/Population, Interest, and Context (PICO/PICo) correctly reported?
- Was the “intervention/interest” of the PICO/PICo correctly reported?
- Was the “comparison” of the PICO/PICo correctly reported?
- Was the “outcome/context” of the PICO/PICo correctly reported?

**Assess**

- Which information sources were used (BMJ Best Practice, Cochrane Library, PubMed, etc)?
- Was a link to research evidence reported?
- Was there an agreement between the information source used and the identified research evidence?

**Appraise**

- Was there an agreement between the identified research evidence and the chosen critical appraisal checklist used?
- Were the questions in the checklist completed?

**Apply**

- Was the application of the research evidence reported (drop-down menu)?
- If reported applied, was this described?

**Audit**

- Were changes in practice evaluated?
- Was the EBP process evaluated?

### Analysis

Descriptive statistics were used to summarize the assessment of students’ EBP skills based on the completed CATs, including frequencies and percentages for categorical variables and mean and range for continuous variables. Associations between correctly reporting the Population, Intervention, Comparison, and Outcome/Population, Interest, and Context (PICO/PICo) elements and finding research evidence were analyzed with the chi-square test with adjustment for repeated measurements [24]. The significance level was set at 5%. Statistical analyses were performed with SPSS Statistics (version 28.0; IBM Corp) [25] and R (R Foundation for Statistical Computing) [26].

### Ethical Considerations

The Norwegian Agency for Shared Services in Education and Research approved the study (project 50425). The students were informed, both orally and in writing, about the purpose of this study and that the data would be treated confidentially. The students agreed to participate in the study and signed a consent form when they created a profile and used the EBPsteps app. The students did not receive any compensation for participating. Students could choose to use the app or a Word document to complete assignments where it was required to produce CATs. The data were securely stored on the research server at HVL.

## Results

### Participants

Among 4 cohorts with OT students, 79.3% (119/150) of students used the EBPsteps app during their fifth semester. The students who used the app produced 240 CATs. In the first cohort (2018), 41 of 47 students produced 73 CATs; in the second cohort (2019), 25 of 30 students produced 53 CATs; in the third cohort (2020), 21 of 33 students produced 43 CATs; and in the fourth cohort (2021), 32 of 40 students produced 71 CATs. The mean number of CATs produced per student was 2, with a range from 1 to 7.

### Step 1: Ask

A need for more knowledge on a clinical problem was reported in 94.6% (227/240) CATs. In 80% (192/240) of the CATs, the type of clinical question was identified using a drop-down menu. A clinical question was formulated in 53.3% (128/240) of the CATs. The “effect of therapy” was the most prevalent clinical question reported (100/240, 41.7%) (Table 1).

All PICO/PICo elements were reported correctly in 10.4% (25/240) of the CATs. Assessing the different PICO/PICo elements separately, the “population” and “intervention/interest” elements were more often correctly reported (187/240, 77.9% and 189/240, 78.8%) than the “comparison” and “outcome/context” elements (44/240, 18.3% and 103/240, 42.9%). This applied to all question types, including when the question had been formulated as a background question (Table 1). In CATs without a clinical question identified, most PICO/PICo elements were incorrectly reported.

**Table 1:** Correctly reported Population, Intervention, Comparison, and Outcome/Population, Interest, and Context (PICO/PICo) elements by type of question in 240 critically appraised topics.

	Population, n (%)	Intervention/interest, n (%)	Comparison, n (%)	Outcome/context, n (%)
Effect of therapy (n=100)	90 (90)	96 (96)	30 (30)	53 (53)
Qualitative (n=27)	25 (93)	25 (93)	N/R^a^	13 (48)
Background (n=64)	55 (86)	52 (81)	11 (17)	32 (50)
Other (n=1) or missing (n=48)	17 (35)	16 (33)	3 (6)	5 (10)

^a^Not relevant.

### Step 2: Assess

In 240 of the CATs, the information source most frequently reported was the Cochrane Library (65/240, 27.1%), followed by CINAHL (43/240, 17.9%), PubMed (36/240, 15%), and Epistemonikos (17/240, 7.1%). In 12.9% (31/240) of the CATs, no information source was reported. Research evidence was identified and linked to in 81.3% (195/240) of the CATs, and the most common type of research evidence identified was systematic reviews (n=85), randomized controlled trials (RCTs; n=51), and qualitative research (n=44).

We observed a positive association between correctly reporting “population” and “intervention/interest” elements of the PICO/PICo and identifying research evidence. Among those correctly reporting the population element, 92.1% (221/240) identified research evidence, compared to 52.1% (125/240) among those that did not report the population element (*P*<.001). Similar findings were observed for the intervention/interest element.

### Step 3: Appraise

A checklist was used in 81.3% (195/240) of the CATs. Of these, the correct checklist was used in 84.6% (165/195) of the CATs; that is, there was agreement between the type of checklist and the research evidence identified (Table 2).

In 98.2% (162/165) of the CATs with a correct checklist, more than 75% of the checklist questions had been answered. Effect estimates from identified research evidence were documented in 27% (21/77) of the checklists for systematic reviews and 36% (15/42) of the checklists for RCTs.

**Table 2:** Type of research evidence identified and agreement with choice of checklist.

Type of research evidence	The agreement between research evidence and checklist, n (%)
Systematic reviews (n=85)	77 (89)
Randomized controlled trials (n=51)	42 (82)
Qualitative research (n=44)	42 (95)
Guidelines (n=4)	2 (50)
Observational studies^a^ (n=11)	2 (18)
The total number of research evidence identified (n=195)	165 (84.6)

^a^Included the following study designs: prevalence (n=1), diagnostic (n=1), cohort (n=3), case-control (n=1), and cross-section (n=5).

### Step 4: Apply

In 39.6% (95/240) of the CATs, it was reported that research evidence was applied in clinical practice. How the research was applied was described sufficiently in only 61% (58/95) of these CATs.

The most common shared decision-making approach reported from a drop-down menu was “identifying preferences” (78/240, 32.5%) and “exploring possibilities” (78/240, 32.5%). Other shared decision-making approaches reported were “presenting choices” (48/240, 20%) and “recommendations” (46/240, 19.2%), “discussing potential” (45/240, 18.8%), “deciding follow-up” (28/240, 11.7%), and “checking recommendations” (24/240, 10%).

### Step 5: Audit

Evaluation of practice changes was reported in 38.6% (93/240) of the CATs. However, details of practice changes were lacking in all these CATs. In 46% (43/93) of the CATs that reported evaluation, it was reported, “did not change practice,” and in 54% (50/93) of these CATs, it was reported that it was “not relevant to change practice.” The EBP process was reported as evaluated in 54.6% (131/240) of the CATs.

## Discussion

### Principal Findings

This study assessed OT students’ EBP skills as reported in the EBPsteps mobile app. We found that students were most often able to perform the EBP steps of “ask,” “assess,” and “appraise” correctly. A positive association was found between formulating the PICO/PICo elements and identifying research evidence. Applying the evidence and evaluating practice change were the least frequently correctly reported steps of the EBP process.

### Comparison to Previous Work

Using data from the EBPsteps app, where students had documented how they followed the EBP process for their clinical question, enabled us to collect objective data on students’ EBP skills. Instruments that objectively measure EBP skills are recommended for acquiring a true reflection of the situation [6,7,11], as opposed to more frequently used self-report assessment tools [6,7]. Although objective assessment is advised, it can be time-consuming to complete and assess [4]. Consequently, self-reported questionnaires are often chosen because of their practicality of administration [9]. Developing an easy-to-administer scoring plan for the EBPsteps app has therefore been important. Against this background, the EBPsteps app can be a valuable contribution to objectively assessing EBP skills related to all 5 steps of the EBP process.

### Ask and Assess

We found a positive association between correctly reporting population and intervention/interest elements of the PICO/PICo and finding research evidence, indicating that completing the PICO/PICo supports students’ ability to retrieve relevant research evidence. These findings align with previous research reporting that a clearly defined question supports students’ ability to retrieve relevant information [27,28]. Furthermore, structuring the question using the PICO/PICo format makes it easier to decide on search terms [2].

### Appraise

The appropriate critical appraisal checklist was chosen in 68.8% (165/240) of the CATs in this study. Nevertheless, few effect estimates were reported in checklists for RCTs and systematic reviews. This might suggest that the students had difficulties interpreting the statistical results. Lack of confidence in interpreting statistical results has previously been reported among health and social care students [29,30]. Acquiring an understanding of effect estimates is necessary when applying EBP [3], and spending more time teaching the understanding of research results to support the students learning and interpretation of research results is recommended [31].

### Apply and Audit

Only about half of the students in this study reported that they applied the research evidence they found, indicating that they struggled using EBP skills beyond the classroom setting, which also correlates with previous research [32,33]. Lehane et al [34] suggest that structural incorporation of EBP during clinical placement, for instance, through easy access to research, EBP mentors, or regular journal clubs, may support the students in applying research evidence. In addition, incorporating assessment of EBP into clinical placement has been shown to influence EBP behavior [5]. In this study, EBP assignments were mandatory in class but not during clinical placement, which may explain why students in this study struggled with the steps of applying and evaluating practice. Providing a mandatory EBP assignment during the clinical placement may support the students in applying EBP and thus also mastering the 2 last steps of the EBP process.

An alternative explanation for why students struggled with the steps of applying and evaluating practice could be that they experienced fatigue or other difficulties using the app. To explore whether other issues influenced students’ skills, we could have further tested the usability of the app. When developing mobile apps for teaching and learning, usability testing is important [35]. Other research methods are necessary to investigate why the 2 last steps of the EBP process were less frequently completed. Future research should include cognitive interview studies (eg, think-aloud methods) and other pilot studies in different populations to evaluate the comprehensiveness and comprehensibility of the app.

### Future Directions

Knowledge of which EBP steps students find most challenging has implications and gives directions for further development of the EBPsteps app and educational instruction in EBP. For example, providing a more comprehensive explanation of how to interpret statistical results in the app could be beneficial. In addition, spending more time teaching statistics and how to read the results seems necessary to improve students’ EBP performance.

A better alignment between what is taught during classes on campus and what students do at placements could also perhaps better facilitate EBP behavior among students. A mandatory assignment where research evidence must be found and discussed with the clinical instructors may help the students apply and evaluate the use of research evidence during clinical placement.

Currently, the EBPsteps app is available only in Norwegian. In the future, we aim to provide user interface translations for several languages [16]. However, we will need to modify options in the app according to the free access resources available in the different countries (eg, databases, guidelines, and e-learning resources). Efforts will be made to find the best solution and to accommodate needs in low- and middle-income countries.

### Methodological Considerations

The main limitation of this study was that we included students from only one profession and from the same educational institution, and thus the generalizability of the results to other institutions and to other health and social care students is reduced. However, the sample consisted of 4 student cohorts from different academic years (from 2018 to 2021; n=119), including 240 CATs. Accordingly, we believe the results from this study can be recognizable and relevant across other populations.

A strength of this study was that the EBPsteps app allowed us to objectively measure the performance of the EBP process using an app that includes all 5 EBP steps. It is recommended that educators select instruments that objectively measure EBP performance [11]. Shaneyfelt et al [6] emphasized the use of online documentation of the EBP steps as a promising approach.

Another strength was that 2 researchers assessed the CATs independently based on a scoring plan, and disagreement was solved through discussion. However, the EBPsteps app and the scoring plan are not validated for assessing EBP, and measurement properties should be examined in future studies.

### Conclusions

We assessed the students’ EBP skills based on how they documented following the EBP steps in the EBPsteps app, and our results showed variations in how well the students mastered the steps. “Apply” and “audit” were the most difficult EBP steps for the students to perform, and this finding has implications and gives directions for further development of the app and educational instruction in EBP. The EBPsteps app is a new and relevant app for students to learn EBP and can be valuable for assessing EBP skills objectively.

## Appendix

Multimedia Appendix 1 Strengthening the Reporting of Observational Studies in Epidemiology (STROBE) checklist.

Multimedia Appendix 2 The scoring plan of EBPsteps.

## Abbreviations

CAT: critically appraised topic
EBP: evidence-based practice
ECTS: European Credit Transfer System
HVL: Western Norway University of Applied Sciences
OT: occupational therapy
PBL: problem-based learning
PICO/PICo: Population, Intervention, Comparison, and Outcome/Population, Interest, and Context
RCT: randomized controlled trial
STROBE: Strengthening the Reporting of Observational Studies in Epidemiology

## Data Availability

The Norwegian data, anonymized by the authors, are publicly and freely available through HVL Open [23].
