# Supplementary material for: Occupational Therapy Students’ Evidence-Based Practice Skills as Reported in a Mobile App: Cross-Sectional Study
Source: JMIR Med Educ. 2024 Feb 21;10:e48507. doi: 10.2196/48507 (PMC10918542; doi:10.2196/48507)
Supplement: Multimedia Appendix 2 [file mededu_v10i1e48507_app2.docx]

**Multimedia Appendix 2.** The scoring plan of the *EBPsteps*

| **EBP steps** | **What was assessed?** | **Descriptions for the assessments** | **Values given for data analysis** |
| --- | --- | --- | --- |
| **Ask** | Was it reflected on the information needs? | Assessed as correct when a need for more knowledge on a clinical problem was presented. | 1 |
|  |  | Assessed as incorrect when only keywords were presented. | 2 |
|  |  | Missing | 99 *** |
|  | Which clinical question was formulated? | Prevalence (How many have a health problem?) | 1 |
|  |  | Cause-aetiology (Why do some people get this problem while others stay healthy?) | 2 |
|  |  | Diagnostics (How can we determine if someone has this problem?) | 3 |
|  |  | Effect of measures (What can be done to prevent or treat the problem?) | 4 |
|  |  | Prognosis (How is the person with the problem?) | 5 |
|  |  | Experiences and attitudes (How are it experienced? What makes it work?) | 6 |
|  |  | Background question, for example: "How to use HABIT in work with children with spastic hemiplegia?" Used question words such as how or which. | 66 *** |
|  |  | Assessed as not completed/incomplete when nothing was written or only keywords were provided; it had to be written as a question. | 99 *** |
|  | Which clinical question was identified (drop-down menu)? | Prevalence | 1 |
|  |  | Cause – aetiology | 2 |
|  |  | Diagnostics | 3 |
|  |  | Effect of measures | 4 |
|  |  | Prognosis | 5 |
|  |  | Experiences and attitudes | 6 |
|  |  | Missing/not identified | 99 *** |
|  | Was there an agreement between the formulated clinical question and the type of question identified from the drop-down menu? | Assessed as correct if there was an agreement between the formulated clinical question and the type of question identified from a drop-down menu (e.g. the formulated clinical question was a question of effect, and the question from the drop-down menu was identified as an effect question). | 1 |
|  |  | Assessed as incorrect if there was no agreement between the formulated clinical question and the type of question identified from a drop-down menu. | 2 |
|  |  | It could not be assessed if a background question was written or a clinical question was not written. | 77 *** |
|  |  | It was not completed if a research question was not identified from the drop-down menu. | 99 *** |
|  | Was the Population of the PICO*/PICo**correctly reported? | Assessed as correct when the Population was related to the type of population and problem area (e.g. elderly with dementia), and/or a problem area (e.g. dementia), and nothing else (e.g. intervention). Different terms/synonyms for the same theme (e.g. dementia and Alzheimer's) approved. | 1 |
|  |  | Assessed as incorrect if other PICO*/PICo** elements were included.  Only age (e.g. elderly) was not sufficient. | 2 |
|  |  | Missing | 99 *** |
|  | Was the Intervention/Interest of the PICO*/PICo** correctly reported? | Assessed as correct when it was about the Intervention/Interest and nothing else. | 1 |
|  |  | Assessed as incorrect if other PICO*/PICo** elements were included. | 2 |
|  |  | Not completed | 99 *** |
|  | Was the Comparison of the PICO*/PICo** correctly reported? | Assessed as correct when it was about the Comparison and nothing else. | 1 |
|  |  | Assessed as incorrect if other PICO*/PICo** elements were included. | 2 |
|  |  | Not relevant for prevalence, cause, prognosis, and experience questions. | 88 |
|  |  | Missing | 99 *** |
|  | Was the Outcome/Context of the PICO*/PICo** correctly reported? | Assessed as correct when it was about the Outcome/Context and nothing else. Included a clear outcome, e.g. ADL function, participation in work, or pain. | 1 |
|  |  | Assessed as incorrect if other PICO*/PICo** elements were included.  Only "effect" was not sufficient as an outcome. | 2 |
|  |  | Missing | 99 *** |
| **Access** | Which information sources were used? | BMJ Best Practice | 1 |
|  |  | UpToDate | 2 |
|  |  | Fagprosedyrer.no | 3 |
|  |  | National Guideline Clearinghouse | 4 |
|  |  | Campbell Library | 5 |
|  |  | Cochrane Library | 6 |
|  |  | Epistemonikos | 7 |
|  |  | Health Evidence | 8 |
|  |  | The Norwegian Health Library search field | 9 |
|  |  | Joanna Briggs Institute EBP | 10 |
|  |  | Otseeker | 11 |
|  |  | PEDro | 12 |
|  |  | AMED | 13 |
|  |  | CINAHL | 14 |
|  |  | Embase | 15 |
|  |  | MEDLINE | 16 |
|  |  | PubMed | 17 |
|  |  | PsycINFO | 18 |
|  |  | SveMed+ | 19 |
|  |  | Other | 20 |
|  |  | Missing | 99 *** |
|  | Was a link to research evidence reported? | Assessed as correct if a research article was included. | 1 |
|  |  | Assessed as incorrect if a report, letter, or similar was included or the research evidence was not included. | 2 |
|  | Was there an agreement between the information source used and the identified research evidence? | Assessed as correct if there was an agreement between the choice of information source and chosen research article. For instance, qualitative questions could be found in Cinahl, Medline, PsycINFO, and/or other general databases. | 1 |
|  |  | Assessed as incorrect if there was no agreement. | 2 |
|  |  | It could not be assessed if a background question was formulated. | 77 *** |
|  |  | Missing | 99 *** |
| **Appraise** | Was there an agreement between the identified research evidence and the chosen critical appraisal checklist used? | Assessed as correct when there was an agreement between the research article and checklist, they included the same study design. | 1 |
|  |  | Assessed as incorrect if there were no agreements. | 2 |
|  |  | It could not be assessed because an article or checklist was not chosen. | 77 *** |
|  | Were the questions in the checklist completed? | Each question in the checklist was assessed individually as correct when completed. | 1 |
|  |  | Each question in the checklist was assessed individually as incorrect when missing. | 2 |
| **Apply** | Was the application of the research evidence reported (drop-down menu)? | Yes | 1 |
|  |  | No | 2 |
|  | If reported applied, was this described? | Assessed as correct if applying the evidence in practice was reported. | 1 |
|  |  | Assessed as incorrect if it was not described how the research was applied. | 2 |
|  |  | Missing | 99 *** |
| **Audit** | Were changes in practice evaluated? | Assessed as correct if changes in practice were evaluated. | 1 |
|  |  | Assesses as incorrect when changes in practice were not evaluated or the reporting of 'not relevant'. | 2 |
|  |  | Assessed when reported 'did not change practice'. | 55 *** |
|  |  | Missing | 99 *** |
|  | Was the EBP process evaluated? | Assessed as correct when reflection was mentioned of one or more steps. | 1 |
|  |  | Assessed as incorrect when no reflection of the steps was mentioned. | 2 |
|  |  | Missing | 99 *** |

* PICO (Abbreviation for Population, Intervention, Comparison, and Outcome). ** PICo (Abbreviation for Population, Interest, and Context). *** These categories were given the value 2 (incorrect) for the analyses in SPSS.
